# Macroscopic Innervation of the Dura Mater Covering the Middle Cranial Fossa in Humans Correlated to Neurovascular Headache

**DOI:** 10.3389/fnana.2017.00127

**Published:** 2017-12-19

**Authors:** Shin-Hyo Lee, Seung-Jun Hwang, Ki-Seok Koh, Wu-Chul Song, Sang-Don Han

**Affiliations:** ^1^Department of Anatomy, Research Institute of Medical Science, Konkuk University School of Medicine, Seoul, South Korea; ^2^Department of Anatomy, Medical Center, University of Ulsan College of Medicine, Seoul, South Korea; ^3^Department of Medical Education, Konkuk University School of Medicine, Seoul, South Korea

**Keywords:** dura mater, middle cranial fossa, middle meningeal artery, nervus spinosus, Sihler’s stain, trigeminal nerve

## Abstract

The trigeminovascular system within the cranial dura mater is a possible cause of headaches. The aim of this study is to investigate macroscopically dural innervation around the middle meningeal artery (MMA) in the middle cranial fossa. Forty-four sides of the cranial dura overlying the skull base obtained from 24 human cadavers were stained using Sihler’s method. Overall, the nervus spinosus (NS) from either the maxillary or mandibular trigeminal divisions ran along the lateral wall of the middle meningeal vein rather than that of the MMA. Distinct bundles of the NS running along the course of the frontal branches of the MMA were present in 81.8% of cases (*N* = 36). Others did not form dominant nerve bundles, instead giving off free nerve endings along the course of the MMA or dural connective tissue. The distribution of these nerve endings was similar to that of the course of the frontal, parietal and petrosal branches of the MMA (11.4%). The others were not restricted to a perivascular plexus, crossing the dural connective tissues far from the MMA (6.8%). These findings indicate that the NS generally travels alongside the course of the frontal branches of the MMA and terminates in the vicinity of the pterion.

## Introduction

Neuroanatomical studies have shown that the trigeminal nerve provides sensory innervation to the intracranial dura mater (O’Connor and van der Kooy, 1986; Strassman et al., 2004; Tomaszewska et al., 2015). Activation of dural sensory fibers is regarded as pivotal for the generation of the pain experienced in some types of headaches, including migraine (May and Goadsby, 1999; Schueler et al., 2013). Typically 15%–20% of the general population suffers from migraine, which is a recurrent, unilateral pulsating headache of moderate-to-severe intensity (Bolay et al., 2002; Lundblad et al., 2015). The pathogenesis of migraine is generally explained by activation of meningeal nociceptors followed the stimulation of the meningeal artery or venous sinuses (Liu et al., 2008; Schueler et al., 2014). While there is still debate over the initiating events in migraine, it is widely believed that the headache pain could arise from activation of the trigeminovascular system in meningeal tissues so as to cause vasodilation (Shevel, 2009; Schueler et al., 2013).

The innervation of the cranial dura mater has been investigated with a focus on structures in the perivascular region such as the middle meningeal artery (MMA) and dural venous sinuses (Hoskin et al., 1999; Liu et al., 2008; De Felice et al., 2010; Huang et al., 2012; Wang et al., 2014; Lundblad et al., 2015). Several previous reports support the presence of the nervus spinosus (NS), which comprises meningeal branches from the maxillary and mandibular trigeminal divisions as a dense plexus along the MMA in the middle cranial fossa (Messlinger et al., 1993; Strassman et al., 2004; Lv et al., 2014; Schueler et al., 2014; Tyburski et al., 2017). In contrast, few studies have macroscopically investigated the trajectory of nerve fibers over long distances of the human cranial dura.

The purpose of the present study was to determine the dural innervation of the entire middle cranial fossa by pan-neuronal staining using Sihler’s method. The findings of this study establish the anatomical basis and variability in the trigeminovascular system of the dura mater housing the important meningeal vessels in humans.

## Materials and Methods

### Subjects

Forty-four cranial dura maters were obtained from 24 formalin-embalmed human cadavers that had been donated to Konkuk University, Ulsan University and Hanyang University in South Korea. The donors comprised 18 males and 6 females who were aged 73.1 ± 9.8 years (mean ± SD) at death. Before they died, the donors signed documents agreeing to their participation in the body donation program of the applicable medical school and the use of their body for clinical studies. The format of the document was consist with the Korean law entitled “Act on Body Donation for Medical Education”. This study was undertaken in accordance with the principles outlined in the Declaration of Helsinki.

**Figure 1:**
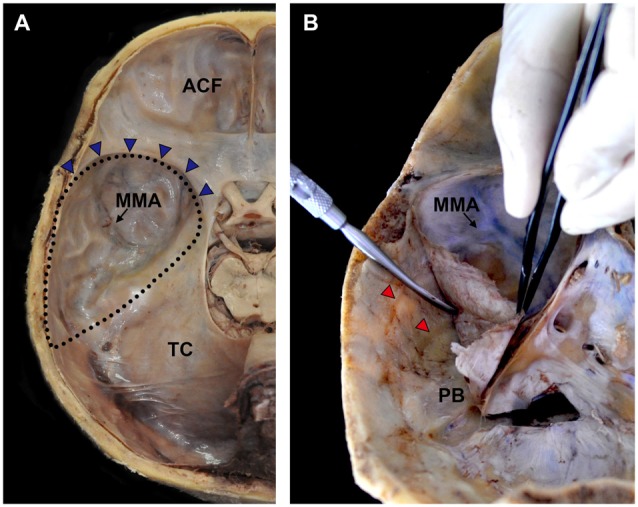
Procedures for harvesting the cranial dura mater covering the middle meningeal fossa. **(A)** The brain was extracted to expose the middle cranial fossa. **(B)** The cranial dura mater was gently detached using an elevator so as to completely obtain the periosteal layer. ACF, anterior cranial fossa; MMA, middle meningeal artery; PB, petrous body; TC, tentorium cerebelli; blue triangles, sphenopalatine sinus; dotted lines, territory of the middle cranial fossa; red triangles, grooves for the MMA.

The calvarium of the neurocranium was dissected using a saw, and the brain was extracted to expose the skull base (Figure 1A). The dura mater is the outmost layer of the intracranial meninges that consist of inner meningeal and external periosteal layers (Kemp et al., 2012). The periosteal layer was detached carefully from the base of the middle cranial fossa using an elevator so as to preserve the nerve fibers between the meningeal and periosteal layers (Figure 1B).

### Sihler’s Stain

The harvested cranial dura mater was immersed in 10% formalin for several weeks. The fixed specimen was washed under running tap water for about 1 h before being immersed in 3% aqueous potassium hydroxide solution for maceration. The duration of maceration varied depending on the degree of blood congestion in the venous sinus in order to prevent laceration of the dura mater. After decalcification in Sihler’s solution I (one volume glacial acetic acid, one volume glycerin and six volumes 1% w/v aqueous chloral hydrate), the specimen was washed under running tap water for about 1 h and stained with Sihler’s solution II (one volume stock Ehrlich’s hematoxylin, one volume glycerin, and six volumes 1% w/v aqueous chloral hydrate) for 1–2 days. The stained specimen was washed under running tap water for about 1 h and then destained in Sihler’s solution I under continuous observation. This applications of Sihler’s method resulted in the nerve fibers being stained dark purple by hematoxylin, which meant that they contrasted with the connective tissue of the dura mater that had been destained rapidly. After destaining, the specimen was neutralized with 0.05% lithium carbonate solution and then subjected to a series of glycerin solutions with increasing concentrations.

### Data Analysis

To obtain focused photographs of the specimen, slits were made in the region of the sphenoparietal sinus, petrous body (PB) and medial side near to the cavernous sinus due to the concavity of the middle cranial fossa. The distance of the main bundles of nerve fibers from the wall of the MMA was evaluated at the intracranial origin of the MMA and the bifurcation point of the frontal and parietal branches. The entire length of the MMA in the middle cranial fossa, the bifurcation point of the frontal and parietal branches of the MMA, the location and number of the main bundle of the nerve fibers crossing the wall of the MMA, and the number of origins of the nerve bundle were also measured. Statistical comparisons were performed using standard software (version 18.0, SPSS for Windows, SPSS, Chicago, IL, USA).

## Results

### Relationship between the NS and MMA

**Figure 2:**
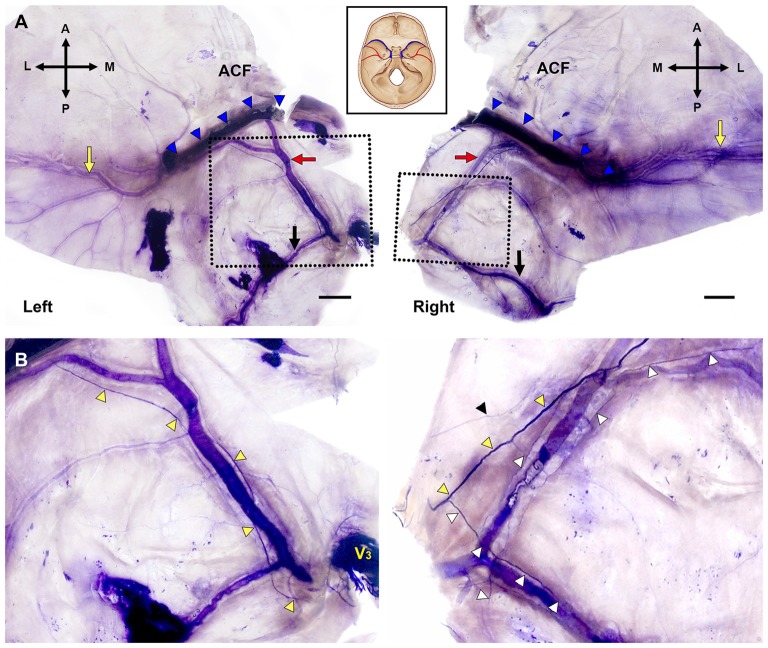
Bilateral macroscopic views of the dura mater of the skull base and temporal region in the same specimen (Bars = 1 cm). **(A)** Flattened dura mater with broad areas containing the MMA (A, anterior; L, lateral; M, medial; P, posterior; black arrows, parietal branch of the MMA; blue triangles, sphenopalatine sinus; red arrows, the frontal branches of the MMA; yellow arrows, distal parts of the frontal branches of the MMA in the temporal region). **(B)** Magnified views of the dotted boxes in **(A)**. The main bundle of the nervus spinosus (NS; yellow triangles) ran along the course of the frontal branches of the MMA crossing the arterial wall. The NS initiated posterior (left) or anterior (right) to the intracranial origin of the MMA. V_3_, mandibular division of the trigeminal ganglion; black triangle, tiny nerve fibers apart from the main bundle of the NS; white triangles, collateral branches from the main bundle of the NS.

**Figure 3:**
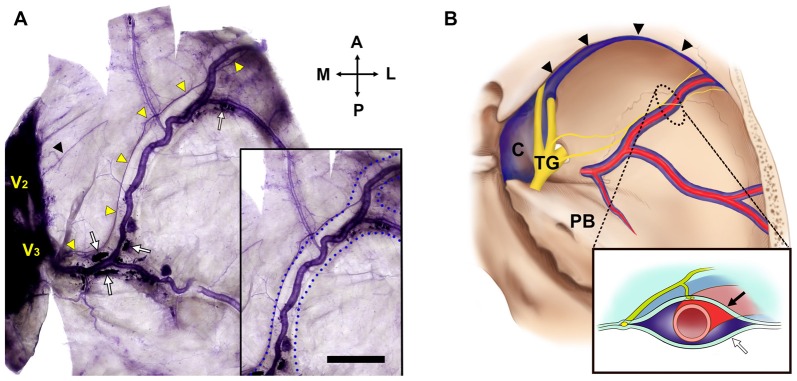
The NS and the middle meningeal vein (Bar = 1 cm). **(A)** The projection of the NS was usually adjacent to the wall of the middle meningeal vein (box) at a certain distance from the wall of the MMA. V_2_, maxillary division of the TG; black triangle, a free nerve ending from the region of V_2_; blue dotted lines, lateral walls of the middle meningeal vein; white arrows, blood clots in the middle meningeal vein; yellow triangles, main bundle of the NS from V_3_. **(B)** Topographic schematics of the NS (yellow), MMA (red) and middle meningeal vein (blue). Lined box is a crossed view of the dotted area. C, cavernous sinus; TG, trigeminal ganglion; black arrow, internal meningeal layer of the cranial dura mater; black triangles, sphenoparietal sinus; white arrow, external periosteal layer of the cranial dura mater.

**Figure 4:**
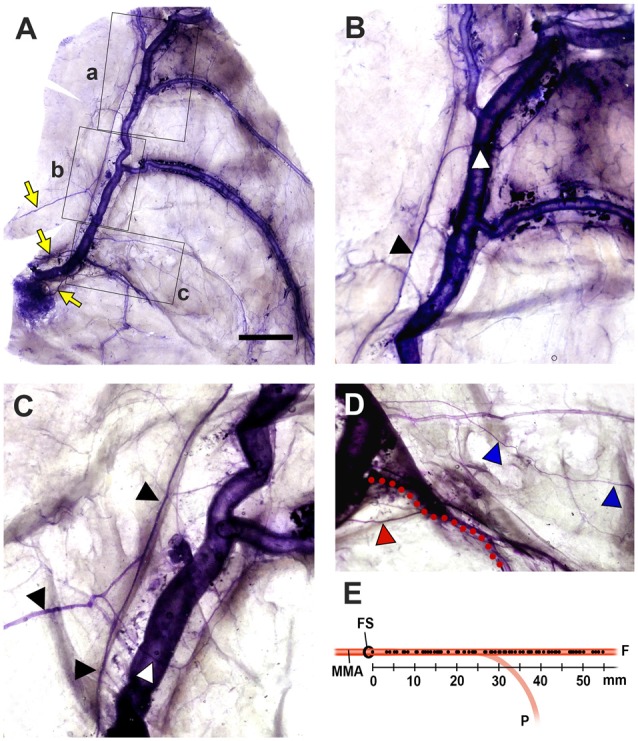
Spatial relationships between the NS and the MMA (Bar = 1 cm). **(A)** The NS originated from three different meningeal branches (yellow arrows), and exhibited significant pruning. **(B–D)** Magnified views of the regions of a,b and c (lined boxes). Arborized nerve fibers (white triangles) from the main bundle of the NS (black triangles) crossed the MMA and projected to the opposite wall of the middle meningeal vein. The petrosal branch of the MMA (red dots) was projected by a distinct meningeal branch (red triangle). A tiny collateral fiber (blue triangles) terminated in the dural connective tissues as free nerve endings that did not reach the main meningeal vessels. **(E)** The crossing point of the NS to the wall of the MMA. This crossing point (dots on the MMA) was evenly distributed of over the entire MMA, indicating that the NS could be sensitized anywhere within the middle cranial fossa. (F, the frontal branches of the MMA; FS, foramen spinosum; P, the parietal branch of the MMA).

The MMA entered the cranial cavity through the foramen spinosum and ramified into frontal, parietal and petrosal branches within the middle cranial fossa (Chmielewski et al., 2013). Distinct nerve fibers of the NS, meningeal branches from the mandibular and/or maxillary divisions of the trigeminal ganglion (TG), initiated their projection medial to the entrance of the MMA into the skull base. Nerve bundles of the NS terminated at a confluence of the middle meningeal vein and the sphenoparietal sinus, and did not reach the region of the anterior cranial fossa (ACF) and distal frontal branches of the MMA in the temporal region (Figure 2). These bundles generally coursed alongside the frontal branches of the MMA at a certain distance from the wall of the artery, parallel to the lateral margin of the middle meningeal vein (Figure 3). Arborized nerve fibers crossed the wall of the MMA and traveled along the opposite wall of the middle meningeal vein. Nerve bundles of the NS were separated from the vascular wall of the MMA anteriorly or posteriorly, by 4.6 ± 3.2 mm at the intracranial origin of the MMA and 1.7 ± 0.5 mm at the bifurcation point of the frontal and parietal branches of the MMA. The NS could originate from multiple locations and some of these nerve fibers exhibited a complicated trajectory (Figure 4). The distance from the intracranial origin of the MMA to where it crossed the NS was 27.9 ± 16.0 mm. The nerve fibers crossed the MMA an average of 1.9 ± 1.4 times. The even distribution of the crossing point indicated that the NS crossed the wall of the MMA randomly (Figure 4E). The measured parameters of the spatial relationships between the MMA and the NS are listed in Table 1.

**Table 1:** Measured parameters of spatial relationships between the nervus spinosus (NS) and the middle meningeal artery (MMA).

Parameter	Values (mm)
MMA length in the MCF	52.3 ± 5.9
FP bifurcation point	20.0 ± 13.7
NS crossing point	27.9 ± 16.0
Number of NS crossings	1.9 ± 1.4
Number of NS origins	1.2 ± 0.5

*Data are mean ± SD values. FP, frontal and parietal branches of the MMA; MCF, middle cranial fossa*.

### Innervation Patterns Referred to the MMA

**Figure 5:**
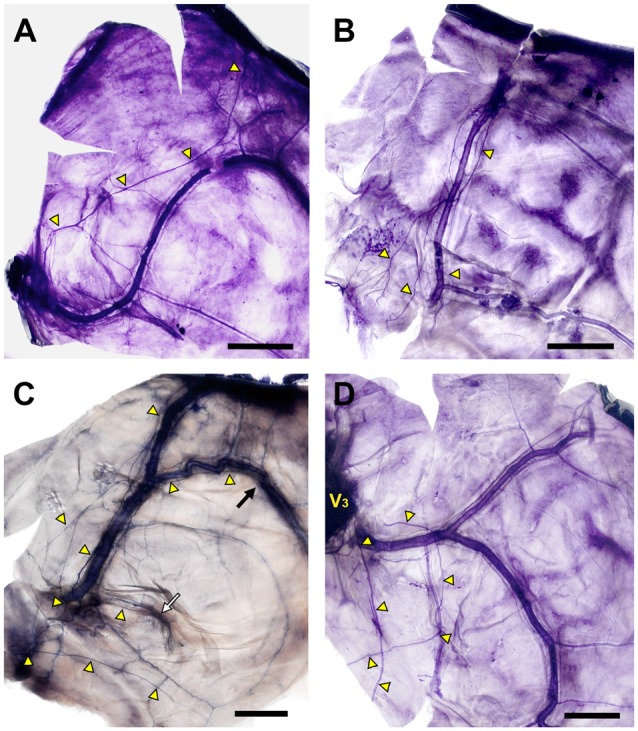
Diverse morphology of the innervation of the NS (yellow triangles; Bars = 1 cm). **(A)** The main bundle of the NS was clearly separated from the course of the MMA. **(B)** The NS formed a plexus at the intracranial origin of the MMA, and converged gradually at the frontal branches of the MMA. **(C)** Meningeal branches of the trigeminal nerve dispersed to the frontal, parietal (black arrow) and petrosal (white arrow) branches of the MMA. **(D)** Meningeal branches from V_3_ projected posteriorly irrespective of the course of the MMA.

Axonal trajectories of the NS were generally adjacent to the region of dural vessels, but, atypical innervation patterns also appeared. The NS generally projected along the course of the MMA, but was clearly separated from the lateral margin of the middle meningeal vein in a few cases (Figure 5A). Some specimens showed ambiguous dominant bundles of the NS (Figures 5B,C) or a nonvascular trajectory of nerve fibers to the dural connective tissue (Figure 5D).

**Figure 6:**
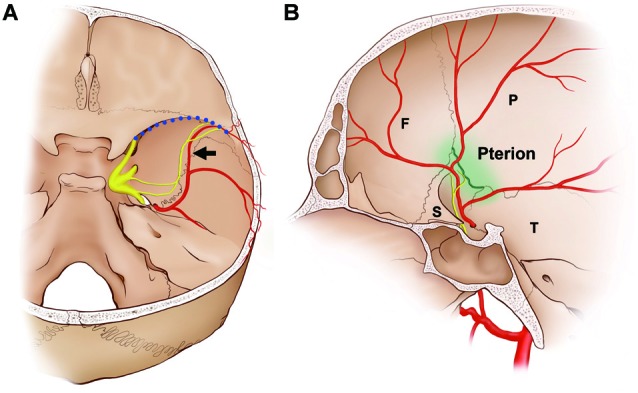
Schematics of the dominant distribution of the NS. In approximately 80% of cases, the NS terminated at the intersection of the frontal branches of the MMA (black arrow) and the sphenoparietal sinus (blood dotted line). **(A)** Posterior view of the middle cranial fossa. **(B)** Medial view of the middle cranial fossa. The interface with the frontal branches of the MMA and the sphenoparietal sinus coincides with the pterion (green line), which is the region where the frontal (F), parietal (P), sphenoid (S) and squamous parts of the temporal bones (T) join together.

The projection patterns of the NS on the 44 sides of the dura mater of the middle cranial fossa were classified according to the trajectories of the nerve fibers. Nerve fibers projected to the course of the frontal branches of the MMA to form distinct nerve bundles sending collaterals in 81.8% of cases (*N* = 36; Figure 6). The remaining innervation patterns did not follow the course of the frontal branches of the MMA. Arborized fibers from the meningeal origin without dominant bundles were distributed to all branches of the MMA equally in 11.4% of cases (*N* = 5). In 6.8% of cases the NS not restricted to a perivascular plexus, crossing the dural connective tissues far from the MMA (*N* = 3).

## Discussion

The pathogenesis of migraine is a matter of ongoing discussion, but close relationships between activation of meningeal sensory fibers of the trigeminal nerve and dural vasodilation/vasoconstriction are thought to underlie the pain experienced in migraine (Kowacs et al., 2004; Strassman et al., 2004; Olesen et al., 2009). Previous neuroanatomical studies showed that the MMA in the dura mater along the floor of the middle cranial fossa is innervated by the NS originating from the mandibular and maxillary trigeminal divisions (Lv et al., 2014; Schueler et al., 2014). The present study has provided more detailed macroscopic information about the course of these meningeal sensory fibers overlying the entire middle cranial fossa in humans. Consistent with findings in experimental animals, the present study found that the nerve bundles of the NS in humans usually coursed alongside the MMA. A particularly notable observation was of several meningeal nerve fibers running along the lateral margin of the middle meningeal vein instead of the MMA. To the best of our knowledge, this characteristic of the NS trajectory has not been reported previously, which might be due to previous descriptions of the morphology of the NS being spatially restricted to nearby the TG or covering only small areas of the MMA microscopically.

In more than 80% of the present cases, the NS ran alongside the frontal branches of the MMA, and terminated at the intersection of the MMA and the sphenoparietal sinus, which is an anterolateral territory of the middle cranial fossa (Figure 6). The frontal branches of the MMA that crossed the floor of the skull base passed through the lateral margin of the sphenoparietal sinus, and were directed laterally to supply the dura mater of the frontotemporal region. The region in which the NS terminated and where the frontal branches of the MMA exited the anterolateral territory of the middle cranial fossa coincided with the pterion, which is where the frontal, sphenoid, parietal and squamous parts of the temporal bones meet (Oguz et al., 2004; Schwartz et al., 2008). Before reaching the pterion, the frontal branches of the MMA and the nerve bundle of the NS ran alongside the floor of the middle cranial fossa parallel to the sphenosquamosal suture, which is the posterior border of the greater wing of the sphenoid bone. Shimizu et al. (2008) found that the MMA pierces a bony tunnel that is an elongation of the middle meningeal groove located on the temporal side of the sphenoid bone just beneath the pterion in 75.8% of 78 sides. This property of the MMA inside the pterion means that there is a risk of extradural hematoma when a fracture occurs. Ma et al. (2012) reported that the pterion overlapped the frontal branches of the MMA in 68% of 152 sides. This pterion junction has been used as a common extracranial landmark for microsurgical approaches in this region, and it corresponds to the anterolateral fontanelle on the neonatal skull that disappears approximately 3 months after birth (Aydin et al., 2010). The formation of TG during the early stage of embryo development (O’Rahilly and Müller, 2007) and the postnatal closure of the pterion might allow extensive communication of extracranial information and intracranial meninges housing important vessels in protecting the brain. Pioneering researches using retrograde neural tracing support the presence of a connection between the intracranial NS and extracranial nociceptive fibers via calvarial sutures (Schueler et al., 2013, 2014; Zhao and Levy, 2014). The pterion could be a practical landmark for the frontal branches of the MMA and the NS during surgical approaches in the temporal region and skull base.

The main limitation of the present study is that only a part of the network of meningeal nerves can be identified using Sihler’s method due to hematoxylin favoring myelinated axons (Mu and Sanders, 2010). Schueler et al. (2013) investigated the NS of humans using electron microscopy examinations, and found that two-thirds of them comprised unmyelinated axons. Slow Aδ and fast Aβ neurons of the dural afferent may play a role in the stimulus afferent pathway due to their higher mechanosensitivity and chemosensitivity (Strassman et al., 2004; Messlinger et al., 2012).

The present study has revealed the macroscopic courses of the NS in the human dura mater. There appear to be close relationships among the NS, the frontal branches of the MMA, and the pterion, which is a typical landmark utilized in neuroanatomy. The NS generally travels alongside the course of frontal branches of the MMA at a certain distance from the wall of the artery, and terminates in the vicinity of the pterion. A detailed understanding based on macroscopic observations of the nerve’s course with reference to the meningeal vessels along the floor of the middle cranial fossa is expected to be useful in the fields of neurosurgery and clinical neurology.

## Author Contributions

S-HL carried out the experiments, drafted the article and illustrated. W-CS, S-JH and S-HL interpreted data. W-CS, K-SK and S-DH revised the manuscript for intellectual content. All authors read and approved the final manuscript.

## Conflict of Interest Statement

The authors declare that the research was conducted in the absence of any commercial or financial relationships that could be construed as a potential conflict of interest.
